# Immunoinformatics and Biophysics Approaches to Design a Novel Multi-Epitopes Vaccine Design against *Staphylococcus auricularis*

**DOI:** 10.3390/vaccines10050637

**Published:** 2022-04-19

**Authors:** Roba Attar, Eid A. Alatawi, Faris F. Aba Alkhayl, Khloud Nawaf Alharbi, Khaled S. Allemailem, Ahmad Almatroudi

**Affiliations:** 1 Department of Biology, Faculty of Science, University of Jeddah, Jeddah 21959, Saudi Arabia; rmattar@uj.edu.sa; 2 Department of Medical Laboratory Technology, Faculty of Applied Medical Sciences, University of Tabuk, Tabuk 71491, Saudi Arabia; eid.alatawi@ut.edu.sa; 3 Department of Medical Laboratories, College of Applied Medical Sciences, Qassim University, Buraydah 51452, Saudi Arabia; ffabaalkhiel@qu.edu.sa (F.F.A.A.); khloud.n.alharbi@gmail.com (K.N.A.); k.allemailem@qu.edu.sa (K.S.A.); 4 Department of Pharmaceutical Chemistry and Pharmacognosy, College of Dentistry and Pharmacy, Buraydah Colleges, Buraydah 51418, Saudi Arabia

**Keywords:** antimicrobial resistance, *Staphylococcus auricularis*, multi-epitopes vaccine, epitopes mapping, molecular dynamics simulation

## Abstract

Due to the misuse of antibiotics in our daily lives, antimicrobial resistance (AMR) has become a major health problem. Penicillin, the first antibiotic, was used in the 1930s and led to the emergence of AMR. Due to alterations in the microbe’s genome and the evolution of new resistance mechanisms, antibiotics are losing efficacy against microbes. There are high rates of mortality and morbidity due to antibiotic resistance, so addressing this major health issue requires new approaches. *Staphylococcus auricularis* is a Gram-positive cocci and is capable of causing opportunistic infections and sepsis. *S. auricularis* is resistant to several antibiotics and does not currently have a licensed vaccine. In this study, we used bacterial pan-genome analysis (BPGA) to study *S. auricularis* pan-genome and applied a reverse immunology approach to prioritize vaccine targets against *S. auricularis*. A total of 15,444 core proteins were identified by BPGA analysis, which were then used to identify good vaccine candidates considering potential vaccine filters. Two vaccine candidates were evaluated for epitope prediction including the superoxide dismutase and gamma-glutamyl transferase protein. The epitope prediction phase involved the prediction of a variety of B-Cell and T-cell epitopes, and the epitopes that met certain criteria, such as antigenicity, immunogenicity, non-allergenicity, and non-toxicity were chosen. A multi-epitopes vaccine construct was then constructed from all the predicted epitopes, and a cholera toxin B-subunit adjuvant was also added to increase vaccine antigenicity. Three-dimensional models of the vaccine were used for downward analyses. Using the best-modeled structure, binding potency was tested with MHC-I, MHC-II and TLR-4 immune cells receptors, proving that the vaccine binds strongly with the receptors. Further, molecular dynamics simulations interpreted strong intermolecular binding between the vaccine and receptors and confirmed the vaccine epitopes exposed to the host immune system. The results support that the vaccine candidate may be capable of eliciting a protective immune response against *S. auricularis* and may be a promising candidate for experimental in vitro and in vivo studies.

## 1. Introduction

Antibiotics are medications that are used to completely eradicated or decrease bacteria growth. Antibiotics cannot treat infections caused by viruses [1]. The first antibiotic was penicillin which was discovered in 1928 by Alexander Fleming [2]. The overuse of antibiotics is a major concern as this causes many bacterial infections resistant to antibacterial drugs causing antimicrobial resistance (AMR) [3]. There is a high rate of morbidity and mortality for those pathogens which show resistance to antibiotics. Around 70,000 total deaths are caused due to AMR each year. Due to the over or misuse of antibiotics, bacteria become resistant to antibiotics by making changes in their genetic makeup [4]. To lower the burden of antibiotic resistance, it is essential to make a vaccine that is safe to use and can stop the antibiotic resistance evolution [5]. Resistance against vaccines is very rare and thus could be a more attractive option to manage bacterial pathogens [6]. 

Vaccines are biological formulations used to stimulate and train the host immune system against infectious agents by producing antibodies and cellular immunity [6]. The first vaccine was developed against smallpox by Edward Jenner by using the virus named cowpox [7]. Different approaches are used in the development of good vaccines and are divided into many types [8]. Then, Louis Pasteur developed a vaccine by using the bacillus in its weakened form to treat anthrax [9]. After Pasteur’s efforts, an extensive number of experiments were conducted in the field for developing vaccines against several diseases. Sabin and stalk also developed a good vaccine against poliovirus using the rules of Pasteur vaccinology [10]. A vaccine named Bacillus Calmette Guerin (BCG) was developed against *Mycobacterium tuberculosis* using Pasteur vaccinology [11]. However, there are some limitations of Pasteur vaccinology as it does not apply to the pathogens which constantly show mutations in antigens present on surfaces or those pathogens whose in vitro culturing is not possible [12]. Furthermore, developing a conventional vaccine is costly and requires a lot of time [13]. Due to these hurdles, a reduction in the development of a culture-based vaccine is seen, and new techniques are used to develop a vaccine [14]. In the last few years, numerous advancements in vaccinology have been reported. In modern vaccinology called reverse vaccinology (RV), we determine the antigenic surface proteins from a database of the genome [15,16]. In RV we identify good vaccine candidates by applying different vaccine filters [17,18]. Through RV we developed a vaccine named meningococcal serogroup B (4CMenB) [19]. In this technique, vaccine targets are identified from genomic data of the pathogen and predicted B-cell and T-cell epitopes are used in constructing a good vaccine candidate [19,20].

*Staphylococcus auricularis*, an uncommon coagulase-negative *Staphylococcus* (CoNS), is commonly considered culture contamination. *S. auricularis* is a Gram-positive species of the genus Staphylococcus composed of two or four cocci cells. *S. auricularis* is a popular colonizer of the ear canal. It is not frequently found to be harmful and has only very infrequently been linked to skin and soft tissue diseases [21]. Because it often resides on human skin, it may be capable of causing opportunistic infectious diseases or sepsis, although this is quite rare. Although staphylococci with no coagulase activity may be a normal part of the skin’s flora, these bacteria can cause infection of the skin and soft tissues [22]. It has been observed that *S. auricularis* causes skin and soft tissue infections [23]. Patients who are immunosuppressed or elderly are more likely to contract these infections, and they are often susceptible to antibiotic treatment. Ear canals are a common site of *S. auricularis* colonization [21]. It is one of the many hundreds of species of bacteria that constitute normal human flora and is rarely a cause of disease. *S. auricularis* causes sepsis in newborn babies [23]. *S. auricularis* is cultivated in cultures of abscesses and paronychia [24]. Thus, coagulase-negative *S. auricularis* should not always be recognized as pollutants or typical flora, but rather as causal pathogens [21,25]. Several vaccine design efforts have been conducted against pathogenic species of the *Staphylococci* genus [25,26]. However, the *S. auricularis* genome has not been explored so far for vaccine targets. As no licensed vaccine is available and the bacterium is evolving new antibiotic resistant mechanisms via horizontal gene transfer, therefore, it needs to be managed by developing a safe and effective vaccine for the pathogen [27,28,29,30]. 

## 2. Research Methodology

**Figure 1:**
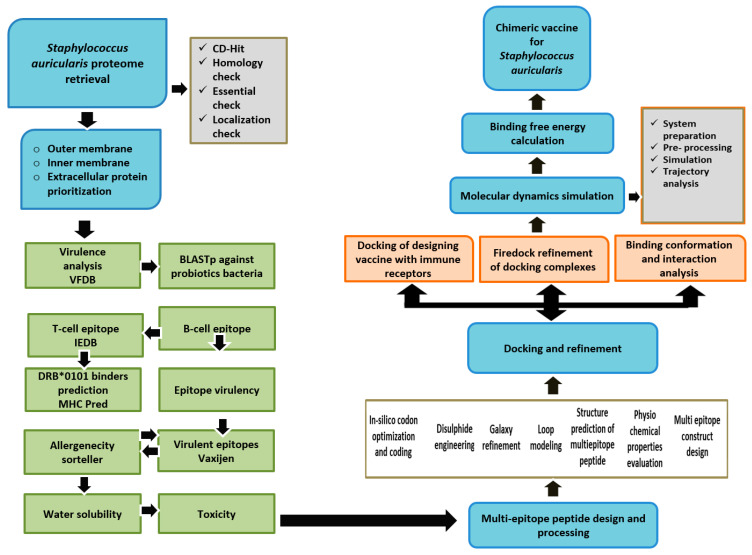
A schematic representation of the approach used to identify vaccine candidates in the *S. auricularis* proteome.

Figure 1 depicts the methods used to build a multi-epitope peptide vaccine for *S. auricularis.*

### 2.1. Bacterial Pan-Genomics, Subtractive Proteomics, and Reverse Vaccinology

Proteomic data of *S. auricularis* was retrieved from NCBI’s genome database [31]. After that, the proteomes were screened for prospective vaccine candidates through bacterial pan-genomics [32,33], reverse vaccinology [34], and subtractive proteomics [35].

### 2.2. Pre-Selection Stage

A bacterial pan-genome analysis (BPGA) technique was used to identify the core proteome of *S. auricularis* [36]. In BPGA analysis, only sequenced genomes were considered and similarity-based clustering was performed at 50%. In BLASTp [31] against human proteome (tax id: 190), the identity of the sequences must be lower than 30%. The non-homologous proteins are ideal candidates for vaccine design as they do not generate auto-immune responses [26,37]. Similarly, to identify pathogenic essential proteins, BLASTp against the DEG database [38] was run, and the proteins that have a sequence similarity score of ≥30% were considered essential [39].

### 2.3. CD-Hit Analysis

Redundant proteins are duplicate copies of genes and, therefore, are not regarded as effective immunological targets [40]. Non-redundant proteins are the best choices for vaccines [41]. All of the pathogen’s non-redundant proteins were anticipated with an online CD-HIT server [42] with a sequence similarity threshold of 50% and all other input values left at defaults settings. When it comes to comparing and clustering protein sequences, CD-HIT has become a popular and commonly utilized server [43].

### 2.4. Subcellular Localization Phase

Subcellular organization of the essential and non-redundant proteome was examined through PSORTb 3.0 [44]. Proteins located at the cell surface of infectious agents play an important role in vaccine design because they interact with hosts and aid in the pathogen’s infectious cycle [45].

### 2.5. Vaccine Candidate’s Prioritization Phase

The pathogenic secretome and exoproteome were further filtered in this stage to find those associated with pathogen pathogenesis and disease progression. The screened proteins present at the pathogen surface or secretory were BLASTp against the virulent factor database (VFDB) [46] for the identification of virulent proteins using sequence specificity of less than 30% and a bit score of greater than 100 [12]. 

### 2.6. Prediction of Immune Cell Epitopes

Bepipred Linear Epitope Prediction 2.0 [47] from the Immune Epitope Data source (IEDB) [48] with a value of 0.5 was used to anticipate the first linear B-cell epitopes for virulent proteins. The B-cell epitopes were then utilized to locate T-cell antigenic determinants using IEDB T-cell antigenic determinants analysis packages to find subsequences that interact with MHC (I and II) alleles [49]. The MHC-I interacting epitopes were predicted using IEDB recommended 2020.09 (NetMHCpan EL 4.4) method considering MHC source species as human and reference HLA allele reference data set while MHC-II epitopes were determined using IEDB recommended 2.22 method. The MHC-I presents epitopes to cytotoxic T-cells while MHC-II alleles represent epitopes to helper T-cells [50,51]. 

### 2.7. MHCPred 2.0 Analysis

MHCPred 2.0 [52] evaluation was performed to determine the binding affinities of screened B-cell derived T-cell antigenic epitopes, and those with an IC_50_ value less than 100 nm for DRB*0101 were considered [53].

### 2.8. Antigenicity, Allergenicity, and Adhesion Probability Prediction

Using VaxiJen 2.0 [54] with a targeted organism set as bacteria and a threshold of >0.4, the antigenicity of predicted epitopes was determined [55]. The accuracy of antigenic targets prediction by VaxiJen varies from organism to organism. For instance, in the case of bacterial antigens, the VaxiJen accuracy is 82%, specificity is 72% and sensitivity is 91% [54]. Allertop 2.0 [56] was used to assess the allergenicity of epitopes [57]. The adhesion properties of the antigenic epitopes were also investigated [58]. As a prospective vaccine target, adhesive proteins let bacteria bind and adhere to host cells, which is essential for infectious pathogenesis. SPAAN was used to estimate the adhesion properties of the antigenic proteins with a baseline range of 0.5 [59].

### 2.9. Multi-Epitopes Peptide Designing

Peptide vaccines have weak immunogenicity that can be solved by integrating immunodominant epitopes to build a multi-epitope peptide vaccine and using suitable adjuvants [60]. GPGPG linkers were utilized to assemble the filtered epitopes into a multi-epitope peptide [61]. The 3D structure of the design was simulated using the 3Dpro of the SCRATCH protein predictor [62]. Galaxy Loop and Galaxy Refine of Galaxy Web were used to simulate the structure’s loops and optimize the structure further [63,64]. Design 2.0 was used to conduct the introduction of the disulfide bonds in the proposed vaccine structure [49]. Design 2.0 uses the Disulfide by Design algorithm which is developed for recognizing protein folds and native geometry [65].

### 2.10. Codon Optimization

The vaccine’s design sequence was reverse translated and optimized for codon usage following the *E. coli* codon usage pattern. The vaccine sequence was reverse translated using Java Codon Adaptation Tool (JCat) service [66,67]. The percent GC concentration and the codon adaptation score (CAI) were used to evaluate the expression rate of the cloned sequence. Ideally, the CAI should be 1. It is recommended that the GC content should be around 30–70 percent because of the high efficiency of transcription and translation.

### 2.11. Docking and Refinement

In this phase, the recombinant vaccine construct was docked with suitable immune receptors. Using a “blind docking” technique, the vaccine constructs natural binding to TLR4 carrying PDB ID: 4G8A, MHC-I (PDB ID: 1I1Y), and MHC-II (PDB ID: 1GKO) was predicted. PATCHDOCK, an online network that permits the docking of molecules based on shape complementarity principles, was used to execute molecular docking [68,69]. The complex type was set to “default” and the clustering RMSD was set to “4.0” [70]. Fast Interaction Refinement in Molecular Docking (FireDock) [71] was utilized to fine-tune the docked complexes instantly. Using Fire dock’s rescoring and refining tools, protein–protein docking solutions could be quickly improved. A low-global-binding-energy complex was chosen for each case by UCSF Chimera [72], which also used intermolecular interaction and binding confirmation selection [73].

### 2.12. Molecular Dynamics (MD) Simulation Assay

In silico technologies, such as molecular dynamics, modeling can be used to study the dynamic behavior of vaccine-immune receptors [74]. Complexes were chosen for the molecular dynamic simulation process based on their global energy values [73]. The research was carried out using AMBER20 simulation software [75] on a 250 ns timescale. The AMBER SANDER module is used to complete the system setup, preprocessing, and production phases. Solvating the compounds into a TIP3P solvation box with padding of 12 Angstroms was the final step. Afterward, the complexes were roasted to 300 K and cooled for 1 ns. As part of this process, each trajectory file was saved at the rate of 10 ns per second. To restrict hydrogen bonds, the SHAKE method [76] was used, while Langevin dynamics was employed to regulate the temperature [77,78]. CPPTRAJ module [79] was used for trajectories analysis. 

### 2.13. Free Energy of Immune Receptors and Vaccine Design

For docked complexes, the MMPBSA.py tool [80] provided in AMBER20 was used to determine the binding free energies. In total, 100 frames were selected from the trajectory of a complete simulation and examined for binding energy. A key goal was to identify the differences in free energy between a solvated and an unsolvated state of complexes.

## 3. Results

### 3.1. Genomes Retrieval of S. auricularis

For developing a vaccine based on multi-epitopes, sequenced genomes of *S. auricularis* were retrieved from the genome database of NCBI. Here, we downloaded eleven genome sequences with both complete and incomplete genomes of *S. auricularis.* The strain size of these pathogens ranges from 2.22 Mb to 2.42 Mb while the GC content is from 37.10 to 37.40. A strain’s type, genome size, and percentage of GC content are presented in Table 1.

### 3.2. Bacterial Pan-Genome Analysis

**Figure 2:**
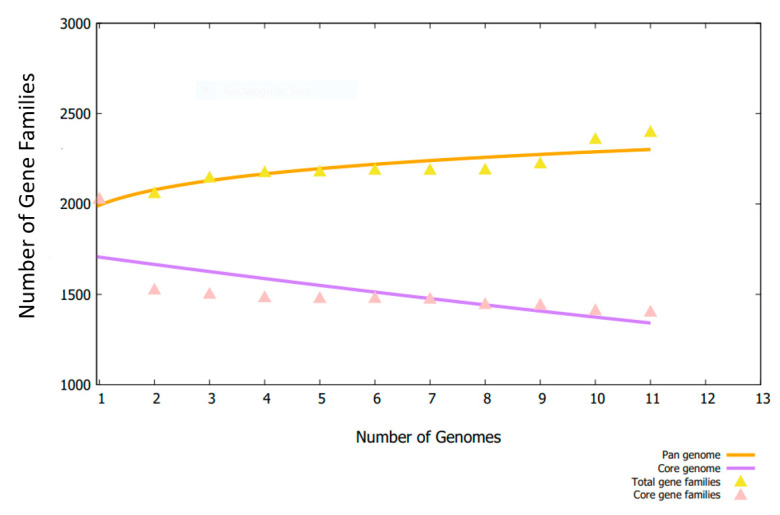
Each strain’s genome size is presented herein. On the x-axis number of genomes is provided while on the y-axis number of gene families in each genome is presented.

**Figure 3:**
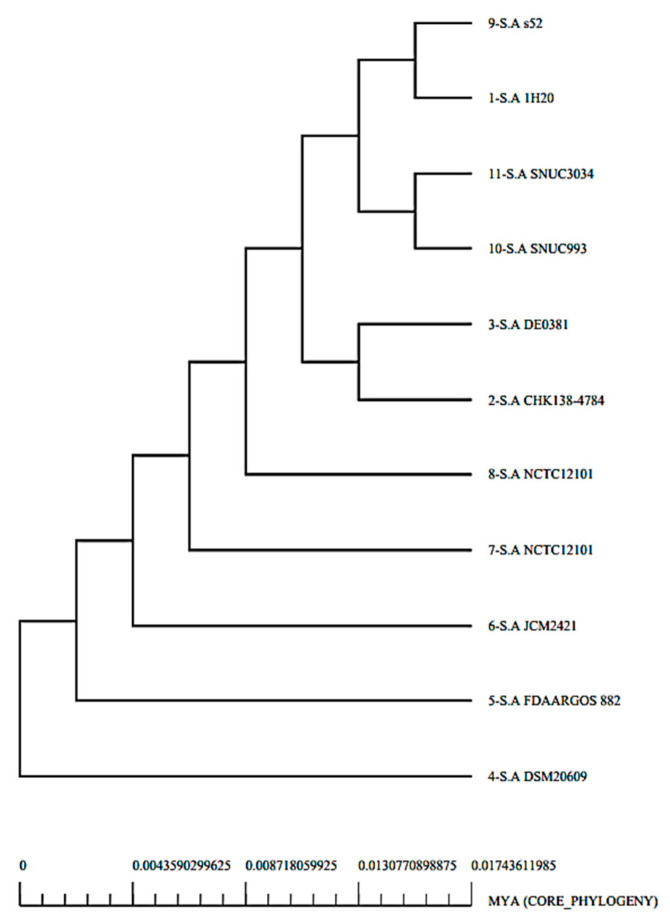
A pan-phylogenetic tree of the genome of *S. auricularis*.

Using bacterial pan-genome analysis, we derived the core genome and accessory genome for downward steps. While the pan-genome includes the sequences of all strains, the core genome contains the sequences that are present in all strains [36]. The accessory genome includes the sequences that occur in only a few strains but are not found in all strains [36]. Unique genes are found only in one strain and are strain-specific, also called singletons. Adjoining or dispensable genes are part of the accessory proteome [36]. Those proteins that are conserved across strains are found in the core genome. Figure 2 shows the genome size for each strain, while Figure 3 presents a pan phylogeny tree of *S. auricularis.*

### 3.3. CD-HIT Analysis and Proteins Subcellular Localization

**Figure 4:**
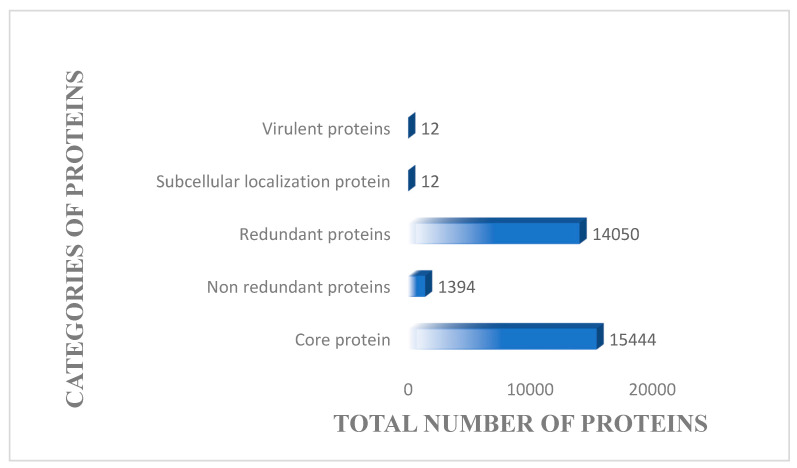
The total number of core proteins, sub-cellular localized proteins, redundant, non-redundant proteins and virulent proteins. The proteins obtained at each step were screened from core proteome of 11 strains of *S. auricularis*.

The core genome of the pathogen is displayed in Figure 4 and is composed of 1394 non-redundant proteins and 14,050 redundant proteins while the total proteome count is 15,444. Attempts were made to remove the redundant proteins since they are duplicates, and thus, not suitable candidates for vaccine development [81]. Surface proteins, as well as proteins in periplasm, extracellular and outer membrane, are easily recognized by the immune system of the host [82]. As shown in Figure 4, a total of 12 proteins have been identified at the pathogen surface.

### 3.4. VFDB Analysis

According to the methodology described in the methodology section, two virulent proteins were identified in the selected 12 extracellular/surface proteins as shown in Table 2. Proteins from virulent organisms serve as ideal vaccine targets since they can stimulate immune pathways and result in more effective, safe immune responses [83,84].

### 3.5. B-Cell Epitopes Prediction 

Two proteins were chosen for epitope prediction after passing through all other essential filters. Thereafter, we will use the IEDB server to predict B-cell epitopes and T-cell epitopes. The first step was to predict B-cell epitopes. For gamma-glutamyl transferase, the total predicted B-cell epitopes were eight and for superoxide dismutase four B-cell epitopes were predicted (Table 3).

### 3.6. MHC-I and MHC-II Epitopes Prediction

The process of predicting T-cell epitopes includes MHC-I and MHC-II binding. These are listed in Table 4. We used the following MHC-I alleles; HLA-A*01:01, HLA-A*01:01, HLA-A*02:01, HLA-A*02:01, HLA-A*02:03, LA-A*02:03, HLA-A*02:06, HLA-A*02:06, HLA-A*03:01, HLA-A*03:01, HLA-A*11:01, HLA-A*11:01, HLA-A*23:01, HLA-A*23:01, HLA-A*24:02, HLA-A*24:02, HLA-A*26:01, HLA-A*26:01, HLA-A*30:01, HLA-A*30:01, HLA-A*30:02, HLA-A*30:02, HLA-A*31:01, HLA-A*31:01, HLA-A*32:01, HLA-A*32:01, HLA-A*33:01, HLA-A*33:01, HLA-A*68:01, HLA-A*68:01, HLA-A*68:02, HLA-A*68:02, HLA-B*07:02, HLA-B*07:02, HLA-B*08:01, HLA-B*08:01, HLA-B*15:01, HLA-B*15:01, HLA-B*35:01, HLA-B*35:01, HLA-B*40:01, HLA-B*40:01, HLA-B*44:02, HLA-B*44:02, HLA-B*44:03, HLA-B*44:03, HLA-B*51:01, HLA-B*51:01, HLA-B*53:01, HLA-B*53:01, HLA-B*57:01, HLA-B*57:01, HLA-B*58:01, HLA-B*58:01) and MHC-II alleles; HLA-DRB1*01:01, HLA-DRB1*03: *04:01, HLA-DRB101,HLA DRB1*04:05, HLADRB1*07:01, HLA,DQA1*03:01/DQB1*03:02,HLADQA1*03:01/DQB1*03:02,HLADQA1*01:02/DQB1*06:02,HLADPA1*02:01/DPB1*01:01,HLADPA1*01:03/DPB1*04:01,HLADPA1*03:01/DPB1*04:02,HLADPA1*02:01/DPB1*05:01,HLADPA1*02:01/DPB1*14:01. The predicted T-cell epitopes are given in Table 4.

### 3.7. Epitope Prioritization Phase

In order to prioritize those epitopes that can be used in a multi-epitope vaccine, various filters such as water-solubility, antigenicity, MHCPred, allergenicity, and toxicity were applied to the selected epitopes.

### 3.8. MHCPred Analysis

MHCPred was used to analyze epitope binding affinity for DRB*0101. Only those epitopes with IC_50_ values less than 100 nm were selected given the prevalence of DRB*0101 in 95% of the population [32]. Table 5 shows those epitopes with IC50 values below 100 nm.

### 3.9. Allergenicity and Antigenicity

Several non-allergic and antigenic epitopes were taken into consideration to stimulate the strongest immune responses. The epitopes that are non-allergic and antigenic are listed in Table 5.

### 3.10. Analysis of Solubility and Toxicity

**Figure 5:**
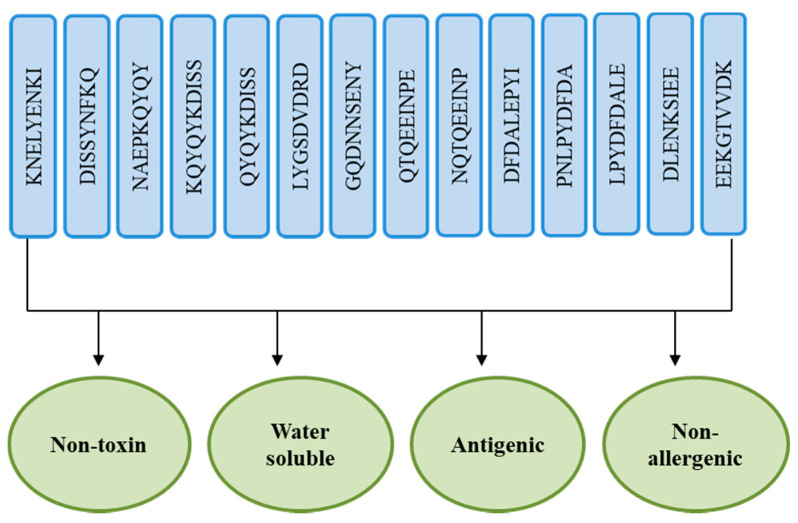
Fourteen shortlisted epitopes for the construction of a multi-epitopes based vaccine.

Epitopes with high solubility were identified using InvivoGen and only non-toxic epitopes were selected using Toxin-Pred [85]. In Table 5, non-allergic, water-soluble, non-toxic, and antigenic epitopes were listed. Figure 5 shows the fourteen shortlisted epitopes and how they will be combined into a multi-epitope vaccine.

### 3.11. Multi-Epitopes Vaccine Designing 

**Figure 6:**
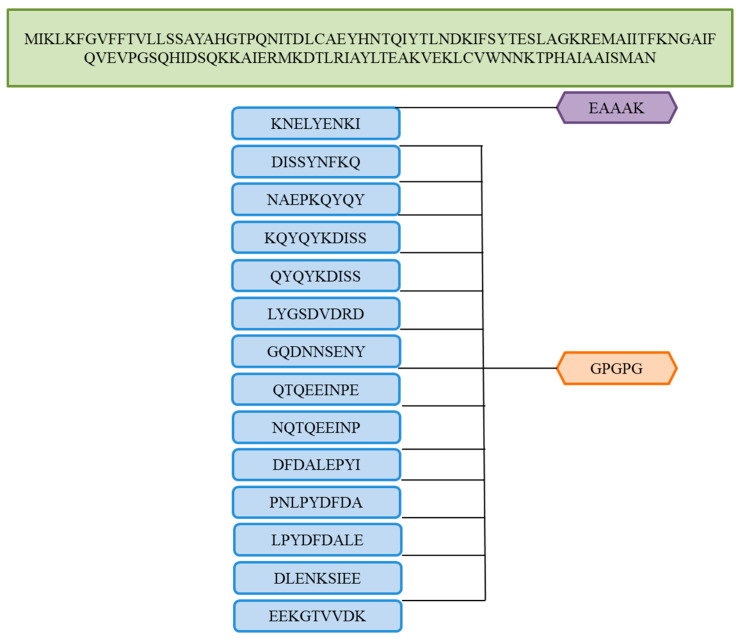
The vaccine construct is described in a schematic diagram. In the orange color, we can see the linker (GPGPG) that is used to link selected epitopes. Green represents cholera toxin B-subunit (adjuvant) and purple represents EAAAK (linker).

The epitopes of the multi-epitope were linked through linkers to allow efficient separation of the epitopes to overcome epitopes’ weak immunogenicity [86]. As well as adding adjuvant molecules to the multi-epitope’s peptide, the immunogenic and antigenic properties of the vaccine were further enhanced [87,88]. A potent interferon stimulator, cholera toxin B-subunit was used as an adjuvant. The schematic representation of the vaccine constructs for multiple epitopes can be found in Figure 6.

### 3.12. Vaccine Structure Modeling 

**Figure 7:**
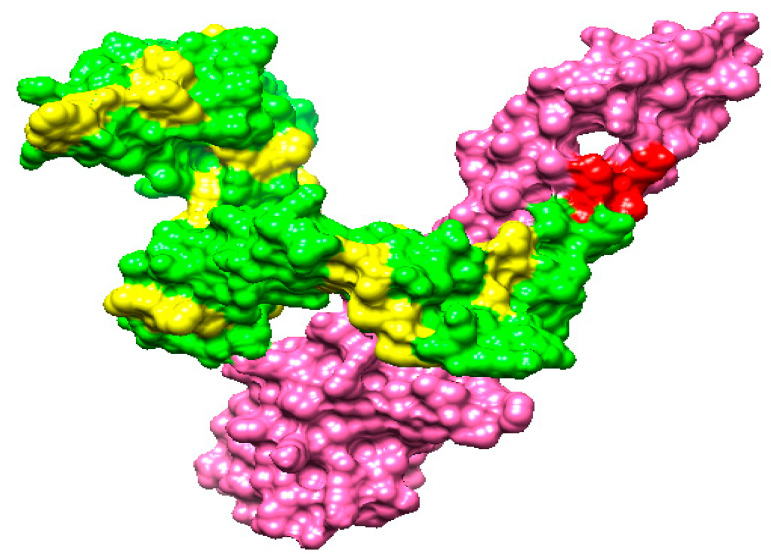
The GPGPG linkers (yellow), cholera toxin B subunit (pink), EAAAK linkers (red), and vaccine epitopes (green) are displayed in the 3D structure of the vaccine construct.

To investigate the interactions between vaccine constructs and immune receptors, as well as the exposed nature of vaccine epitopes, a three-dimensional model of the vaccine construct was developed. At the time, no suitable template existed for vaccine structure modeling, so Ab initio modeling was performed. A 3D model of the vaccine is shown in Figure 7.

### 3.13. Loop Modeling and Refinement

Keeping the structure stable following loops which contain residues; TYR33-ILE38, LYS64-PRO74, GLY139-GLY143, GLY167-GLN173, GLN187-ASP192, ASP207-PRO213, GLU232-PRO236, PRO253-GLU262, GLY280-LEU285, LEU300-SER304, PRO311-LYS321, GLU50-LYS55, GLU100-ASN111, GLN152-ALA159, TYR174-ILE179, ILE193-PRO199, GLY214-SER220, GLU237-GLY242, GLY266-GLY270, PRO286-LEU292, ILE305-GLY310, ARG56-PHE63, GLU160-TYR166, SER180-GLY186, GLY200-VAL206, GLY224-GLY228, ASN243-GLY252, PRO271-TYR275, GLU293-ASP299 to obtain the most refined structure, they were modeled into secondary structure elements.

### 3.14. Disulfide Engineering

**Figure 8:**
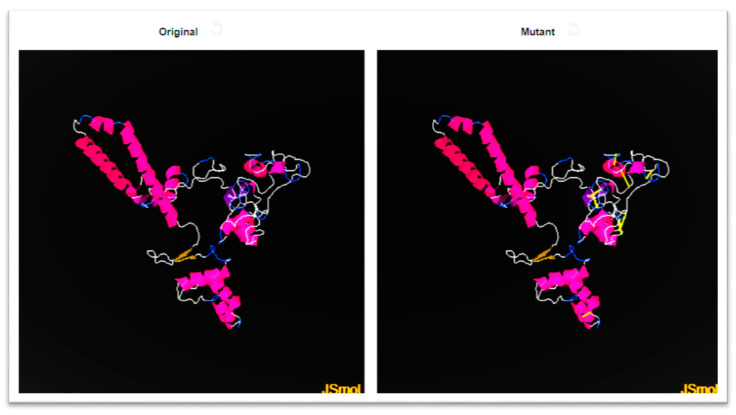
A mutated and wild vaccine construct. Mutated structures show yellow bands where disulfide bonds have been introduced.

Disulfide engineering was applied to the vaccine in order to improve the intermolecular bonding and structural stability of the vaccine. Moreover, it assures that the weaker components of the vaccine are resistant to cellular degradation and impart configurational stability to the vaccine [89]. Cys residues were transformed to cysteine only in groups with a greater energy level (>0 kcal/mol). Cysteine replaces the amino acid residues listed in Table 6, and the cysteine linkages can be seen in Figure 8 as a yellow stick.

### 3.15. Optimizing Codon Sequences

Following the *E. coli* expression system, the reversed translation of the vaccine sequence into a DNA sequence was carried out and then optimized for codon usage. The vaccine has a GC level of 57% and a CAI percentage of 0.92%. Both values indicate a high-expressed sequence [90].

### 3.16. Analysis of Molecular Docking

In order to generate a good immune response, vaccines need to interact effectively with receptors. Through blind docking, we analyze host–receptor interactions with the vaccine construct. Based on the Top 20 docked solutions of vaccines with TLR-4, MHC-I, and MHC-II, are presented in Table 7, Table 8 and Table 9, respectively.

### 3.17. Docked Complexes Refinement

A further refinement was performed on docked complexes to remove false-positive results and select those with the lowest binding energy. Vaccines that bind best with immune receptors will have the lowest binding energy. For MHC-I, solution number 4 was selected due to its low global energy of 4.17 KJ·m^−1^. The global binding energy for solution number 9 was −5.24 KJ·m^−1^ in MHC II. In this case of TLR-4, solution 3 has the lowest global energy of −10.12 KJ·m^−1^ as compared to any other solution. Docked solutions that have been recorded by FireDock are shown in Table 10, Table 11 and Table 12, respectively.

### 3.18. Docked Conformation of Vaccine with Immune Receptors 

**Figure 9:**
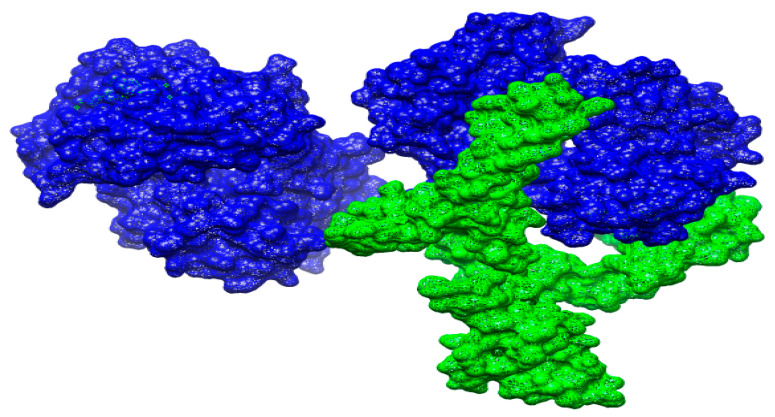
Docking of the vaccine to the MHC-I molecule. The green color shows the vaccine molecule while blue represents MHC-I.

**Figure 10:**
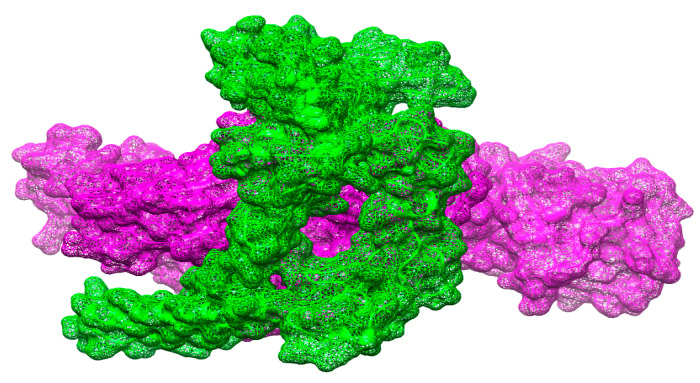
Docking of the vaccine to the MHC-II molecule. The green color shows the vaccine molecule and pink represents MHC-II.

**Figure 11:**
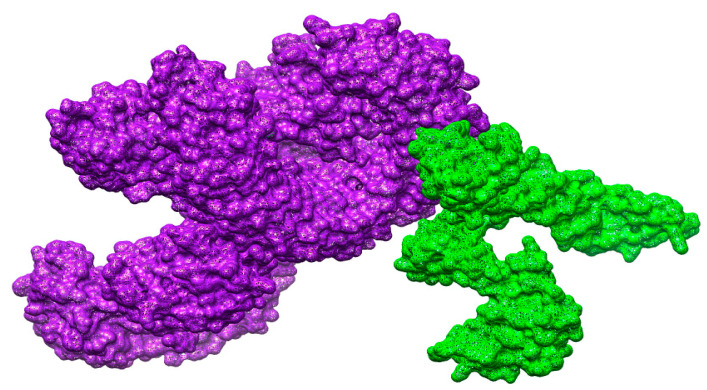
Docking of the vaccine to the TLR-4 molecule. The green color shows the vaccine molecule while purple represents TLR-4.

To evaluate docked configuration of vaccine with immunological receptors, such as MHC-I, MHC-II, and TLR-4, the optimal docked complex for every receptor was visualized, as illustrated in Figure 9, Figure 10 and Figure 11. It was discovered that the vaccines had a strong binding affinity for the receptors, allowing the vaccine’s epitopes to be recognized and processed by the body’s immune system. The vaccine’s epitopes can trigger powerful immunological mechanisms and elicit significant and protective immune reactions are additionally implied by this information.

### 3.19. Interactions of Vaccine to Immune Receptors 

Determining the nature and frequency of interactions between the vaccine and receptors is vital, as they determine the intensity of vaccine–receptor interactions. Between the vaccination and receptors, various forms of interactions were identified, including hydrophilic, hydrophobic, salt bridges, and disulfide bonds. They all play a crucial role in the vaccine’s capacity to maintain its docked conformation with the immunological receptors. To involve the vaccine components, these interactions necessitate the presence of many receptor residues. Table 13 shows these residues.

### 3.20. Molecular Dynamic Simulation

**Figure 12:**
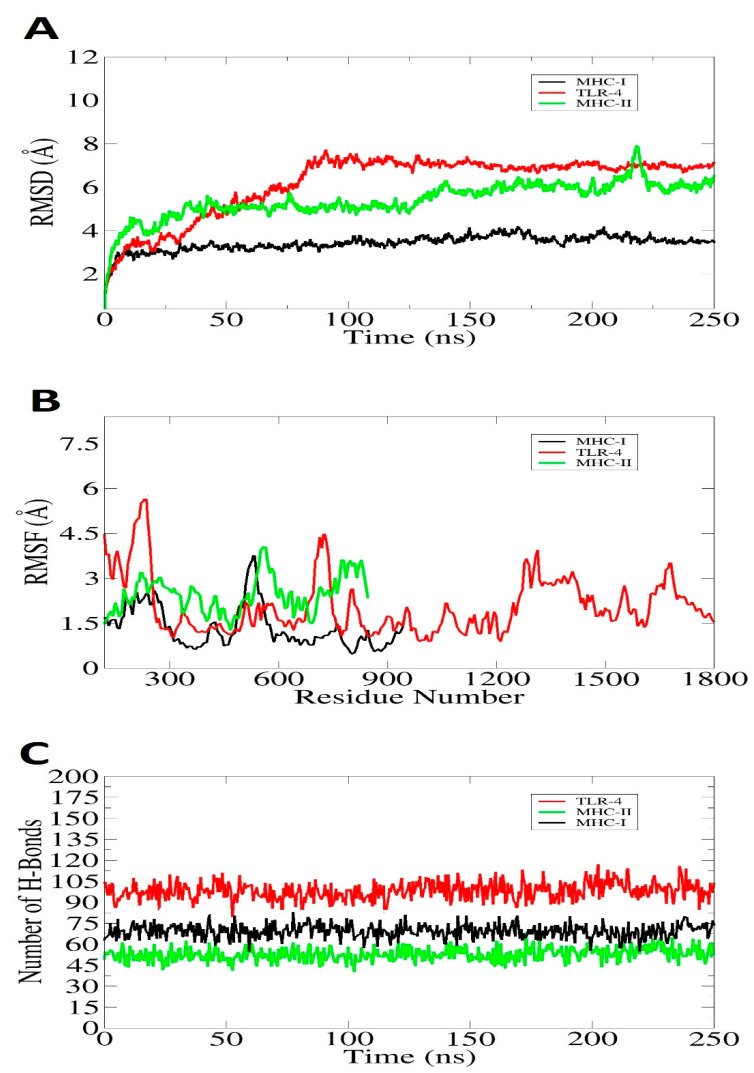
Simulation trajectories analysis via: RMSD (**A**), RMSF (**B**), and number of Hydrogen bonds (**C**).

The molecular dynamics simulation of all atoms examined the dynamic behavior of chosen docked complexes. Based on the carbon alpha atoms, we examined the simulation trajectories using the root-mean-square-deviation (RMSD), root-mean-square-fluctuation (RMSF), and hydrogen bonding. To assess if the vaccination epitopes are expressed to the host immune cells and to better comprehend the dynamic binding stability of the vaccine to receptors, this investigation was essential. The RMSD plot remained stable throughout the study, and no significant structural differences were noted. Due to the multiple loops in the systems, a few minor structural abnormalities were observed. According to Figure 12A, the RMSD plot fluctuates around 4–8 Å during a whole simulation run. In addition, the results of the RMSF showed that the key receptor residues retained stability, with just a few high flexibilities because of many loops in the presence of the vaccine molecules. A large percentage of the system’s residues are below 8 Å, indicating that they are more stable than average (Figure 12B). It was found that the systems were compact, and the secondary structures had a tight shape. These findings are comparable to those of RMSD and RMSF and suggest that the system is stable. H-bonds are formed when an electronegative atom of hydrogen bonds with an electronegative atom of another particle. In the case of electronegative acceptors and donor H-bonds, these bonds are formed. The VMD plugin was used to count and identify the H-bonds that originated in the simulation process, as shown in Figure 12C. While the cut-off distance was shown to be 3 Å for all those H-bonds, a pattern of clustering was shown between TLR4, MHC 1 and MHC 11 receptors, and the peptide vaccine construct model. The complex, however, proves to be strong and has a great affinity for binding to the peptide construct and its corresponding immune receptor.

### 3.21. Estimation of Binding Free Energy

The MM-GBSA and MM-PBSA techniques were used for computing the binding free energy of docked complexes. The fast speed and high efficiency of these two techniques make them highly recognized and reasonable strategies. These estimations were utilized to confirm the binding stability of docked complexes. In MM-GBSA, the total binding free energy of the vaccine-TLR-4 complex is −413.43 kcal/mol, the vaccine-MHC-I complex is −311.01 kcal/mol and for vaccine-MHC-II, the vaccine complex is −270.46 kcal/mol as presented in Table 14. In MM-PBSA the net binding energy is 4–14.23 kcal/mol, −314.55 kcal/mol and −271.82 kcal/mol for vaccine-TLR-4 complex, vaccine-MHC-I complex, and vaccine-MHC-II complex, respectively. Electrostatic and van der Waals energy reveals significant contributions in complexation.

## 4. Discussion

Traditional vaccines made by inactivating or live-attenuating the infectious agent are effective in inducing immunological responses [9]. However, these vaccines could be allergenic and poisonous because of toxic and potent allergic components. While recombinant vaccines (subunit and conjugates) were developed for targeting a particular protein or toxin, the existence of non-antigenic or allergic factors could result in severe toxicity and vaccine failure [91,92]. Bioinformatics resources are being used to develop multi-epitope vaccines employing a reverse vaccinology strategy [93,94]. Immunoinformatics techniques are being used to reduce the time and expense of vaccine design. The multi-epitopes vaccines are designed with antigenicity, non-toxicity, and non-allergenic properties [95].

In the current research, we used various computational methods to determine epitopes from cell wall adherence proteins of *S. auricularis*. The retrieval of proteomic data for *S. auricularis* was carried out from NCBI’s genome database. The prospective vaccine candidates were prioritized through procedures mentioned in the literature. The physicochemical properties were evaluated using web ProtParm software. The transmembrane helices in the selected epitopes were determined using HMMTOP 2.0. The targeted proteins may be proved as efficient vaccine candidates against a variety of *S. auricularis* strains because they promote cell attachment, nutrition acquisition, and escape from host immune responses. The proteins also cleared the antigenicity, allergenicity, toxicity, and immunogenicity checks that are needed for a successful vaccine design. The antigenicity was evaluated using VaxiJen 2.0, Allertop 2.0 was utilized to determine allergenicity. The adhesive properties of selected epitopes were evaluated by SPAAN. The vaccine epitopes were joined together using particular GPGPG linkers to enhance stability and immunogenicity. Linkers are essential for the functional and structural behavior of fusion proteins and the depiction of specific epitopes in the entire design of the vaccine.

Due to the low proportion of epitopes involved, subunit vaccines have low immunogenicity; however, this can be enhanced with an adjuvant. The beta subunit of cholera toxin was employed as an adjuvant to improve immunogenicity in current immunoinformatics research to develop a new multi-epitope vaccine for bacterial infections caused by *S. auricularis* [96]. The vaccines designed through multi-epitopes are generally inadequately immunogenic and need adjuvant coupling. Therefore, vaccine design after adjuvant coupling was examined for antigenic and allergenic effects and found to be antigenic and non-allergic. Homology modeling was applied to assess the 3D structure of the vaccine design, which was then accompanied by molecular docking and MD-simulations to evaluate the stability of the association with its particular TLR-4, MHC-1, and MHC-II immune receptors. A well-known TLR-4 is an immune receptor that triggers reactions against a diverse array of ligands and can activate inherent immunologic reactions by causing the formation of pro-inflammatory mediators in response to challenges with *S. auricularis* [97].

The binding affinities of screened B-cell generated T-cell antigenic factors were determined using MHCPred 2.0. The RMSD and RMSF charts revealed that the vaccine design had a substantial binding affinity for the immune receptor, with minimal deviation. The determination of binding free energy of the TLR4 and multi-epitope vaccine design was carried out using the MMPBSA.py tool. Protein folding and stability depend on disulfide bonding. Furthermore, structural disulfide engineering reduces the range of potential conformations for a particular protein, leading to lowered entropy and enhanced thermostability. The disulfide bond was introduced on the designed vaccine structure using Design 2.0. Despite multi-epitope vaccines being extremely selective to induce a targeted immune response and minimize allergenic responses, in certain situations, the peptide vaccine expressing the modeled epitopes would be slighter immunogenic due to enzymatic degradation in the serum [91].

Purity and stability are two challenges that arise while using peptide-based vaccinations. The most significant problem in peptide vaccine development is incorporating post-translational changes, i.e., glycosylation into recombinant peptides [8]. A computational method for the development and in-silico evaluation of a multi-epitope vaccination against *S. auricularis* targeting the TLR-4 immune receptor is presented in this research. The multi-epitope vaccine developed in this research must first be examined in animal models and clinical trials to comprehend and investigate the immunological responses of the multi-epitope immunization in humans.

## 5. Conclusions and Limitations

Several computer-aided vaccine design techniques have been used in this study to create a multi-epitope vaccine against *S. auricularis*. These techniques include subtractive proteomics, reverse vaccinology, immunoinformatics and several biophysical analyses. Two vaccine targets were used to predict the vaccine epitopes; superoxide dismutase and gamma-glutamyl transferase. The superoxide dismutase has been revealed as a protective antigen against *Campylobacter jejuni* colonization in chickens. The protein is shown to induce high titer antibodies in chicken serum [98]. Similarly, the gamma-glutamyl transferase is effective in inducing protective immune responses against *Helicobacter pylori* by promoting effector helper T-cell differentiation into regulatory T-cells [99]. In choosing the targets for vaccine development, several parameters were considered, such as the presence of proteins in the core proteome, present on the cell surface, non-homologous to the host, probiotic bacteria, and feasibility of experimental analysis. In addition, the epitopes that are used in the vaccine are antigenic, not toxic or allergenic, and they bind tightly to B-cell alleles, as well as T-cell alleles. After being simulated, the constructed vaccine showed excellent binding with the different immune receptors. The simulation of host immunity in response to the vaccine revealed a strong primary, secondary, and tertiary immune response. In light of these findings, it was determined that the vaccine was a good candidate to be tested in vivo for immunity. Our findings and data of the study might accelerate the development of vaccines against *S. auricularis*. We used some pretty tight criteria in selecting subjects for our study, but there are still some shortcomings that need to be addressed in future research. A vaccine’s order of epitopes is not tested for optimal activity. Furthermore, MHC epitopes prediction algorithms are not extensively tested for accuracy.

## Figures and Tables

**Table 1:** *S. auricularis* strains with different statistics.

Organism Name	Strain	Size (Mb)	GC%
*S. auricularis*	FDAARGOS_882	2.22	37.40
*S. auricularis*	NCTC12101	2.22	37.40
*S. auricularis*	JCM 2421	2.26	37.40
*S. auricularis*	DSM 20609	2.20	37.30
*S. auricularis*	NCTC 12101	2.20	37.20
*S. auricularis*	DE0381	2.42	37.40
*S. auricularis*	S52	2.28	37.10
*S. auricularis*	1H20	2.25	37.10
*S. auricularis*	SNUC 3034	2.28	37.10
*S. auricularis*	SNUC 993	2.30	37.10
*S. auricularis*	CHK138−4784	1.68	37.10

**Table 2:** From the exposed proteins, virulent proteins were identified.

Proteins	Subcellular Localization	Bit Score	Sequence Identity
core/231/1/Org1_Gene1151 gamma-glutamyl transferase	Extracellular	244 bits	34%
core/1478/1/Org1_Gene484 superoxide dismutase	Periplasmic	179 bits	43%

**Table 3:** B-cell epitopes for selected proteins.

B-Cell Epitopes	Peptides
core/231/1/Org1_Gene1151 gamma-glutamyl transferase	PDTYDKNELYENKIAGHTQSASNQ
	QNAEPKQYQYKDISSYNFKQ
	LYGSDVDRDSPFFDGSRTKREGDVVK
	KQLDNKLTKKDFQDYEMT
	VNGQDNNSENYLSQE
	NEVNQTQEEINPEGIDN
	SDPNSPNYGEKHKQ
	IDVFKNMGYNVEEKRNDP
core/1478/1/Org1_Gene484 superoxide dismutase	LPNLPYDFDALEPYIDKE
	DLENKSIEEIVANLDSVPED
	TPNSEEKGTVVDKIKEQWGSL
	LKYQNKRPEYIE

**Table 4:** MHC-I and MHC-II epitope prediction by B-Cell epitopes.

MHC-II	Percentile Score	MHC-I	Percentile Score
KIAGHTQSASN	4.3	KIAGHTQSA	0.15
		AGHTQSASN	28
PDTYDKNELYENKI	28	PDTYDKNELY	0.28
		DKNELYENKI	20
KQYQYKDISSYNFKQ	4.8	QYQYKDISSY	0.01
		DISSYNFKQ	4.4
NAEPKQYQYKDISS	28	NAEPKQYQY	0.03
		KQYQYKDISS	7.4
LYGSDVDRDSPFFD	7	DVDRDSPFF	0.41
		LYGSDVDRD	24
		DVDRDSPFFD	9.9
SPFFDGSRTKREGDV	37	FFDGSRTKR	0.17
		SPFFDGSRTK	0.31
		GSRTKREGDV	13
LTKKDFQDYEM	7.2	LTKKDFQDY	0.2
		KKDFQDYEM	2.9
KQLDNKLTKKDFQDY	33	KQLDNKLTK	0.05
		LTKKDFQDY	0.2
GQDNNSENYLSQE	45	GQDNNSENY	0.28
		NSENYLSQE	7.8
NEVNQTQEEINPE	13	EVNQTQEEI	0.1
		NEVNQTQEEI	0.79
		QTQEEINPE	6.3
VNQTQEEINPEGIDN	22	QEEINPEGI	0.63
		VNQTQEEINP	37
SDPNSPNYGEKHKQ	74	NSPNYGEKHK	3.4
		DPNSPNYGEK	3.5
		SDPNSPNYGE	6.8
IDVFKNMGYNV	0.82	DVFKNGYNV	0.34
		IDVFKNMGY	1.4
VFKNMGYNVEEKRND	12	MGYNVEEKR	0.62
		VFKNMGYNV	1.3
		YNVEEKRND	65
PNLPYDFDALEPYI	1.1	DFDALEPYI	1.9
		PNLPYDFDAL	38
LPYDFDALEPYIDKE		LPYDFDALE	1.7
		DALEPYIDKE	14
IEEIVANLDSVPE	3.2	IEEIVANLD	18
		VANLDSVPE	9.3
DLENKSIEEIVANLD	11	DLENKSIEEI	1.4
		SIEEIVANLD	17
TVVDKIKEQWGSL	22	TVVDKIKEQW	0.08
		KIKEQWGSL	0.26
TPNSEEKGTVVDKI	43	EEKGTVVDKI	0.57
		TPNSEEKGTV	0.89
LKYQNKRPEYI	3	YQNKRPEYI	0.21

**Table 5:** MHC-Pred results, antigenicity, allergenicity, solubility, and toxin-pred profiling are considered in shortlisting epitopes.

MHC- Pred	DRB*0101 IC_50_ Score	Antigenicity	Allergenicity	Solubility	Toxin Pred
KNELYENKI	7.768	0.5035			
DISSYNFKQ	57.15	1.5642			
NAEPKQYQY	2.93	1.0100			
KQYQYKDISS	83.37	0.5079			
QYQYKDISS	11.02	0.5677			
LYGSDVDRD	37.24	0.9838			
GQDNNSENY	64.86	1.5275	Non-allergen	Good water solubility	Non-toxin
QTQEEINPE	6.34	1.1296			
NQTQEEINP	22.39	0.9309			
DFDALEPYI	9.38	1.2216			
PNLPYDFDA	18.54	1.0231			
LPYDFDALE	56.36	0.7775			
DLENKSIEE	32.43	1.3709			
EEKGTVVDK	42.27	0.8901			

**Table 6:** List of residues replaced by cysteine.

Sequence Number	Amino Acid	Sequence Number	Amino Acid	Chi3	Energy	Sum B-Factors
7	GLY	34	HIS	67.21	2.37	0
15	SER	24	GLN	88.65	5.88	0
18	TYR	23	PRO	74.33	4.57	0
209	ASP	218	ASN	122.68	5.37	0
216	GLN	220	SER	122.93	4.95	0
225	PRO	229	GLN	113.02	3.01	0
238	GLY	245	THR	110.79	5.25	0
285	LEU	288	ASP	106.06	3.04	0
301	GLU	305	ILE	106.28	3.68	0

**Table 7:** Vaccine solutions docked for the top 20 MHC-I vaccines.

Solution No	Score	Area	ACE	Transformation
1	20132	3445.10	405.18	−0.44 0.33 1.63 99.21 38.67 29.15
2	19844	3938.00	311.82	−0.92 0.91 0.60 22.26–27.15 9.21
3	19282	2805.20	378.13	−2.47–0.49 3.11–18.74 12.00 37.06
4	19272	3180.70	371.95	−0.55–0.04 2.27 92.68 70.24 41.81
5	18218	2781.40	283.06	−0.95 0.40–3.05 42.02 21.11 3.99
6	17986	2731.90	493.30	−1.97 0.09 0.80 2.95 47.68 44.62
7	17968	3816.80	300.18	−3.01 1.42 1.28–14.91–40.50–6.83
8	17940	2845.80	427.92	−2.81 0.37–2.54 46.23–29.01 36.16
9	17936	3052.60	86.01	−2.91–0.90 2.92–3.45 14.79 55.77
10	17934	3182.80	498.19	2.21 0.23–1.45 52.65–6.49 5.15
11	17904	2907.60	493.90	−0.97 0.93 0.88 33.16–26.75 13.49
12	17688	2908.80	139.47	2.77–0.08–0.59 64.71 52.46 56.37
13	17674	2832.70	465.57	0.17–0.26 2.33 14.30–1.73–34.33
14	17502	2380.50	477.76	−0.85–0.07 0.54 22.31 1.39 66.68
15	17168	2920.20	471.12	2.24 0.17–0.67 28.98 58.30–17.67
16	16922	2557.70	389.82	−0.39 1.28 2.15 47.56 2.78–27.80
17	16866	3903.90	336.25	−1.45 0.79 1.08 27.04–33.05 26.94
18	16826	2201.00	447.60	−0.07–0.17–2.61–7.26 71.44 30.42
19	16782	3966.20	232.52	−0.83–0.06–1.50 2.64 41.63 19.99
20	16776	3039.60	442.93	−0.33 0.92–2.63–9.19 69.57 23.36

**Table 8:** Vaccine solutions docked for the top 20 MHC-II vaccines.

Solution No	Score	Area	ACE	Transformation
1	20570	3565.40	271.97	−2.17 –0.39 –2.54 108.70 52.17 53.60
2	20382	3218.70	−25.73	–1.94 –0.37 2.71 87.91 105.99 58.18
3	19196	3055.90	213.63	1.68 0.11 1.94 111.16 36.90 –54.35
4	18992	3096.60	143.82	0.14 –0.74 –1.31 39.48 110.23 –5.13
5	18592	2957.80	177.57	1.48 –0.90 1.70 138.56 40.32 –13.49
6	18488	3716.80	101.29	–1.83 –0.24 2.67 94.90 105.50 59.00
7	17942	2977.00	343.89	−0.38 –0.06 0.02 117.98 14.99 –19.99
8	17780	2450.90	395.91	0.82 0.11 –1.88 125.52 53.90 –48.68
9	17736	3020.90	355.92	1.52 0.18 1.93 115.95 39.51 –55.51
10	17612	4129.70	451.99	1.30 –0.16 1.77 110.37 47.24 –52.75
11	17540	2462.60	404.36	−1.18 0.51 –1.31 117.85 80.56 6.15
12	17310	2888.70	355.39	−3.03 0.48 –1.16 105.35 115.94 –6.39
13	17232	2316.70	477.49	−2.65 –0.81 –2.79 76.34 64.59 43.98
14	17136	2466.10	424.10	−2.22 0.14 –0.88 133.03 42.01 49.26
15	17096	2801.00	425.08	1.22 –0.46 1.74 94.04 58.55 –31.78
16	17074	2166.70	249.61	−3.05 0.31 0.14 88.68 108.92 35.75
17	16878	2952.00	489.28	−1.91 0.06 2.00 123.27 28.22 11.90
18	16832	2913.60	347.82	−0.77 –0.12 1.64 149.12 75.77 44.31
19	16808	2452.30	270.53	−0.36 0.38 –1.74 81.35 107.07 26.55
20	16784	3289.80	440.55	−1.76 –0.04 1.19 102.77 90.46 58.32

**Table 9:** Vaccine solutions docked for the top 20 TLR4 vaccines.

Solution No	Score	Area	ACE	Transformation
1	20378	3608.40	443.05	3.04 −0.64 1.00 −38.53 51.32 −52.20
2	20200	3636.60	420.46	2.82 0.21 −0.74 −50.55 43.33 −63.93
3	19730	3020.20	148.51	2.05 0.19 2.66 −27.29 20.10 −80.32
4	19420	3354.30	328.45	−0.55 0.18 −2.68 −51.45 45.12 17.38
5	18960	3190.80	253.67	−1.65 −0.25 −0.03 −36.03 32.54 −32.10
6	18942	3010.20	418.13	−0.74 −0.23 1.98 −0.03 −42.39 −0.40
7	18908	3164.90	159.14	2.21 0.07 0.42 −62.64 46.15 −9.60
8	18654	2702.20	457.75	2.89 0.54 −1.86 −6.36 25.96 8.73
9	18428	2381.70	407.50	0.19 0.20 2.02 26.92 22.52 −8.30
10	18120	2812.80	185.16	−0.76 −0.73 −1.94 −27.68 −21.12 14.81
11	17936	2628.00	−81.00	−0.16 −0.13 2.43 39.24 44.49 −72.70
12	17846	2217.50	249.64	1.79 −0.07 −1.37 30.39 33.31 −87.83
13	17816	2887.90	356.04	−0.07 −0.95 2.66 46.97 46.18 −40.96
14	17780	2755.00	288.75	1.14 0.48 1.45 4.04 −19.81 −27.50
15	17736	2691.70	452.35	0.96 1.15 −3.01 −7.89 −5.87 −90.50
16	17596	3641.90	295.19	−2.03 −0.23 −1.28 0.76 48.92 7.22
17	17324	2388.50	215.65	0.78 −0.70 −2.12 16.08 33.32 −89.40
18	17006	3484.90	158.11	1.30 −0.00 2.52 10.41 6.75 −92.90
19	17004	2233.20	413.44	1.14 0.40 1.64 5.01 −17.91 −26.25
20	16972	2286.50	443.84	0.89 −0.21 −2.41 −72.64 11.88 −109.83

**Table 10:** MHC-I-vaccine FireDock solutions. Energy is measured in KJ·m^−1^ for each term listed below.

Rank	Solution Number	Global Energy	Attractive VdW	Repulsive VdW	ACE	HB
1	4	4.17	−31.16	19.84	11.47	−5.30
2	5	6.15	−0.68	0.00	−0.75	0.00
3	3	19.06	−22.15	22.69	19.85	−0.33
4	8	52.95	−14.47	49.91	11.17	−3.19
5	10	455.54	−48.39	574.75	33.65	−7.69
6	6	743.07	−36.55	965.01	16.58	−8.90
7	9	1611.59	−20.94	2061.10	−6.63	−5.60
8	1	3726.53	−70.15	4711.56	12.36	−7.89
9	2	8261.20	−123.35	10537.72	22.74	−27.64
10	7	10978.99	−103.14	13898.94	7.02	−12.71

**Table 11:** FireDock solutions for MHC-II vaccine. In each of the terms below, the unit of energy is KJ·m^−1^.

Rank	Solution Number	Global Energy	Attractive VdW	Repulsive VdW	ACE	HB
1	9	−5.24	−4.69	3.43	0.93	0.00
2	8	19.99	−16.03	44.33	3.41	−2.16
3	4	87.59	−20.16	93.00	12.23	−0.25
4	3	266.04	−22.59	342.76	5.64	−2.57
5	5	323.56	−29.38	438.65	9.04	−1.39
6	6	411.02	−30.17	555.41	6.83	−4.74
7	1	869.72	−55.34	1144.92	15.69	−6.62
8	7	1648.44	−43.84	2070.98	19.56	−4.58
9	10	3313.72	−59.53	4183.44	30.29	−4.02
10	2	3450.81	−76.67	4459.19	9.78	−10.96

**Table 12:** Solutions of TLR-4 vaccine by FireDock. For each term below, KJ·m^−1^ represents the equivalent amount of energy.

Rank	Solution Number	Global Energy	Attractive VdW	Repulsive VdW	ACE	HB
1	3	−10.12	−30.06	23.12	5.72	−1.33
2	10	−3.55	−36.23	23.67	16.26	−1.65
3	8	10.03	−0.01	0.00	0.05	0.00
4	6	17.42	−19.98	5.60	15.01	−0.67
5	9	28.73	−25.53	41.89	14.33	−3.45
6	5	31.17	−27.30	8.09	18.01	−3.54
7	1	33.43	−21.47	21.40	22.06	−2.42
8	4	183.74	−33.69	303.96	2.38	−4.28
9	2	508.87	−22.98	645.31	15.89	−7.35
10	7	1460.30	−73.92	1947.38	−1.23	−6.66

**Table 13:** Interaction between receptor residues and vaccine molecules.

Vaccine Complex	Interactive Residues
MHC-I	ASN 42, ALA 59, ASN 131, ASP 276, ASN 218, ASN 110, GLU 104, GLY 182, GLN 187, GLY 240, GLU 307, HIS 34, ILE 138, ILE 234, LYS162, LYS 5, LEU 13, MET 58, PHE 150, PRO 170, PRO 269, TYR 148, TYR 33, THR 49, VAL 206, VAL 71
MHC-II	ASP 43, ASP 178, ALA 159, ASP 290, ALA 53, ARG 56, GLY 203, GLY 268, GLU 247, GLN 77, GLY 200, GLN 175, HIS 20, LEU 52, LYS 84, PRO 251, SER 195, SER 81, TYR 223, TYR 166, TYR 287
TLR-4	ASP 28, ALA 59, ARG 94, ALA 119, ASP 144, ASN 302, ASP 320, CYS 30, CYS 107, GLY 184, GLY 256, GLY 296, ILE 45, ILE 68, ILU 133, LYS 162, LYS 315, LEU 200, PHE 10, THR 99, TRP 109, TYR 275VAL 71, VAL 206

**Table 14:** Binding free energies shown in kcal/mol.

Energy Parameter	TLR-4-Vaccine Complex	MHC-I-Vaccine Complex	MHC-II-Vaccine Complex
MM-GBSA			
VDWAALS	−280.47	−214.36	−194.85
Electrostatic	−172.96	−146.39	−105.61
Delta G solv	40.00	49.74	30.00
Delta Total	−413.43	−311.01	−270.46
MM-PBSA			
VDWAALS	−280.47	−214.36	−194.85
EEL	−172.96	−146.39	−105.61
Delta G solv	39.20	46.20	28.64
Delta Total	−414.23	−314.55	−271.82

## Data Availability

The data presented in this study are available within the article.
